# Habit and embodiment in Merleau-Ponty

**DOI:** 10.3389/fnhum.2014.00542

**Published:** 2014-07-25

**Authors:** Patricia Moya

**Affiliations:** Philosophy Department, Universidad de los AndesSantiago, Chile

**Keywords:** habit, Merleau-Ponty, embodiment, pre-reflective knowledge, Gallagher, Zahavi

## Introduction

Merleau-Ponty (French phenomenological philosopher, born in 1908 and deceased in 1961) refers to habit in various passages of his *Phenomenology of Perception* as a relevant issue in his philosophical and phenomenological position. Through his exploration of this issue he explains both the pre-reflexive character that our original linkage with the world has, as well as the kind of “understanding” that our body develops with regard to the world. These two characteristics of human existence bear a close relation with the vision of an embodied mind sustained by Gallagher and Zahavi in their work *The Phenomenological Mind: An Introduction to Philosophy of Mind and Cognitive Science*. Merleau-Ponty uses concepts like those of the *lived* or *own* body and of *lived* space in order to emphasize, from a first-person perspective, the co-penetration that exists between subject and world.

Gallagher and Zahavi have regained the experience of phenomenology, especially that of Merleau-Ponty and Sartre, to contribute to the development of the cognitive sciences. Via the phenomenological approach to the reality of habit, a new understanding of the body becomes possible for us, such that it becomes characterized “as subject, as experiencer, as agent,” and at the same time we can understand “the way the body structures our experience” (Gallagher and Zahavi, 2008). Additionally, the idea of a pre-reflexive understanding is conceived of by these authors as a way for refuting those introspective or reflexive explanations that derive from the Cartesian tradition and which are promoted by certain contemporary authors (see, for instance, Dennett, 1991; Price and Aydede, 2005).

In this article I propose to explain the role that habit plays in the phenomenology of Merleau-Ponty and the use that Gallagher and Zahavi make of his theory in their work on cognitive science. The goal of these authors in the work mentioned above goes beyond that of an analysis of habit: they want to demonstrate that “phenomenology addresses issues and provides analyses that are crucial for an understanding of the true complexity of consciousness and cognition,” and thereby reverse the contemporary situation where this perspective is frequently absent from current debates (Gallagher and Zahavi, 2008). For this reason, the neuroscientific community could know a more unified perspective of human behavior. The habit explanation given by Merleau-Ponty shows a kind of body *knowledge* that cannot be exclusively understood by neurological processes.

This paper could provide the neuroscientific community with a more unified perspective of human behavior. The explanation given by Merleau-Ponty of the habit shows a kind of corporeal *knowledge* which cannot be only clarified by neurological processes.

## Embodied consciousness

According to Merleau-Ponty, there is no hard separation between bodily conduct and intelligent conduct; rather, there is a unity of behavior that expresses the intentionality and hence the meaning of this conduct. In habits, the body adapts to the intended meaning, thus giving itself a form of embodied consciousness. Indeed, for our author, corporeal existence constitutes a third category that unifies and transcends the physiological and psychological (cf. Merleau-Ponty, 2012; see also Merleau-Ponty, 1964).

For this reason, Gallagher and Zahavi hold that the philosophy of Merleau-Ponty incorporates the body as “a constitutive or transcendental principle, precisely because it is involved in the very possibility of experience” (Gallagher and Zahavi, 2008). From the perspective of cognitive science, they propose that “the notion of an embodied mind or a minded body, is meant to replace the ordinary notions of mind and body, both of which are derivations and abstractions” (Gallagher and Zahavi, 2008). They note that, by way of confirming the priority of the body, the biological fact of the vertical position of the human body has consequences in the perception and action of the person (cf. Gallagher and Zahavi, 2008)^1^.

## Habit and understanding of the world

Merleau-Ponty explains that the *lived* human body relates to a space that is also *lived*, i.e., that is already incorporated into the world understood as the horizon of its coming to be. According to this view, habit presupposes a form of “understanding” that the body has of the world in which it carries out its operations. An *operant intentionality* (*fungierende Intentionalität*) is established with the world, using the terminology of Husserl (see Merleau-Ponty, 2012). That is, the corporeal subject is inserted into a world that provokes certain questions or problems that must be resolved. Therefore, one can speak of a motivation on the part of the world, although not of a necessity, because the response is not mechanical or determined^2^. Between the movement of the body and the world, no form of representation is established, but rather the body “adapts” to the invitation of the world (cf. Merleau-Ponty, 2012). On the basis of this idea of Merleau-Ponty, Gallagher and Zahavi add: “The environment calls forth a specific body-style so that the body works with the environment and is included in it. The posture that the body adopts in a situation is its way of responding to the environment” (Gallagher and Zahavi, 2008). These affirmations are supported by studies that show that the nervous system does not process any information that does not proceed from corporeality (cf. Zajac, 1993; Chiel and Beer, 1997).

Habit bears a direct relation to this form of *dialog* between environment and subject. Its role is to establish in time those behaviors or forms of conduct that are appropriate for responding to the invitations of the environment. Merleau-Ponty, in establishing the etymological root of the term “habit,” notes that the word *have* states a relation with what has been acquired by the subject as a possession, which in the case of the body is conserved as a dynamic corporeal scheme (Merleau-Ponty, 2012). Thanks to habit, the person establishes appropriate relations with the world that surrounds him or her without needing any prior reasoning, but rather in a spontaneous or immediate way (cf. Merleau-Ponty, 2012). Gallagher and Zahavi also refer to this form of pre-reflexive understanding, relating it to proprioception, i.e., those sensations by which we know where and how our body is, and that are in our consciousness in a tacit manner (cf. Gallagher and Zahavi, 2008; see also Legrand, 2006)^3^. This perspective allows them to distance themselves from representationalist interpretations—for instance, those of Damasio (1999) and Crick (1995), among others—that do not recognize that perception is meaningful in itself (cf. Gallagher and Zahavi, 2008)

We can speak of an engagement of body and world, in which a relation is created that serves as the basis or ground for the rest of the actions of the subject, and which permits him or her to be especially “at home,” comfortable, able to move in an oriented way in a given space (cf. Talero, 2005; Merleau-Ponty, 2012). Just as Gallagher and Zahavi note, this connection with the world does not only mean knowing the physical environment in which the body is situated, “but to be in rapport with circumstances that are bodily meaningful” (Gallagher and Zahavi, 2008).

## Habitual and actual body

According to Merleau-Ponty, the situated character of the person explains that there is, at the same time, a “general” existence as well as an existence that is linked with the effectiveness of action, and which we can call “personal.” Being anchored in the world makes the person renounce a part of his or her protagonism because he or she already possesses a series of habitualities. In this counterpoint between the general and the protagonistic, there occurs “this back-and-forth of existence that sometimes allows itself to exist as a body and sometimes carries itself into personals acts” (Merleau-Ponty, 2012). Merleau-Ponty distinguishes the habitual body—that of general and pre-reflexive existence—from the actual—that of personal and reflexive existence—understanding that both always co-penetrate each other. He explains that in the behaviors of mentally ill or brain damaged persons the nexus between the habitual and the actual body are broken (cf. Merleau-Ponty, 2012). In these cases, the person can reproduce certain habitual movements, but not those that require an actual understanding of the situation. For instance, a person can perform movements like touching his or her nose with a hand, but cannot respond to an order to touch the nose with a ruler. In contrast, in the non-pathological subject there is no rupture between either form of movement, since he or she is able to grasp this analogous form of movement toward the nose that the sick person cannot achieve (cf. Merleau-Ponty, 2012). The healthy person is able to come and go from the habitual to the actual. He or she is able to readjust the habitual to the actual. The world appears to the healthy subject as unfinished, offering him or her a set of possibilities such that experience “is shaped by the insistence of the world as much as it is by my embodied and enactive interests” (Gallagher and Zahavi, 2008).

## The primacy of practical action and the grasping of meaning

In the linkage of the subject with the world, effective, practical action has primacy. In the words of our philosopher, there is always “another self that has already sided with the world, that is already open to certain of its aspects and synchronized with them” (Merleau-Ponty, 2012; see also Talero, 2005). Merleau-Ponty frequently expresses the close relation between body and world with the term “inhabit,” as referring to that which is known by the body and which translates into a knowledge of what to do with an object without any reflexion coming in between (cf. Merleau-Ponty, 2012)^4^. Gallagher and Zahavi corroborate these affirmations with research that relates perception and kinesthesia, as well as with the “enactive theory of perception” (see Varela et al., 1991). In their studies, they show that perception is not a passive reception of information, but instead implies activity, specifically, the movement of our body^5^.

Merleau-Ponty explains that habitual behavior arises on the basis of a set of situations and responses that, despite not being identical, constitute a community of meaning (cf. Merleau-Ponty, 2012). This is possible because the body “understands” the situation in the face of which it must act. For example, in the case of motor habits, such as dancing, the body “traps” and “understands” movement. This is explained by the fact that the subject integrates certain elements of general motility that permit him or her to grasp what is essential to the dance in question and perform it with an ease that is expressed in the mastery of the body over the movements (cf. Merleau-Ponty, 2012). The ability acquired “will lead to performance without explicit monitoring of bodily movement; the skill becomes fully embodied and embedded within the proper context” (Gallagher and Zahavi, 2008). This corporealization of habit agrees fully with the idea of Merleau-Ponty that the body is a correlate of the world: “Habit expresses the power we have of dilating our being in the world, or of altering our existence through incorporating new instruments” (Merleau-Ponty, 2012). Gallagher and Zahavi take from Merleau-Ponty this non-automatic understanding of habitual acts that, despite not requiring an express intentionality, nonetheless form part of the operative intentionality that was mentioned at the beginning of this article (cf. Gallagher and Zahavi, 2008). Citing Leder, they state: “A skill is finally and fully learned when something that once was extrinsic, grasped only through explicit rules or examples, now comes to pervade my own corporeality. My arms know to swim, my mouth can at last speak the language” (Leder, 1990).

Gallagher and Zahavi are able, over the course of their book, to demonstrate the error of that naturalism that defends objective natural science as the only legitimate manner of understanding the mind (cf. Gallagher and Zahavi, 2008; one example, among others, of this posture is found in Sellars, 1963 and in Dennett, 1991).^6^ In contrast, they hold that there is a reciprocal influence between science and phenomenology, just as Varela et al. (1991) understood it via his neurophenomenology based on aspects of the phenomenology of perception of Merleau-Ponty (cf. Gallagher and Zahavi, 2008; see also Gallagher, 1997).

### Conflict of interest statement

The author declares that the research was conducted in the absence of any commercial or financial relationships that could be construed as a potential conflict of interest.

## Footnotes

^1^Cf. also the works that these authors cite by Straus (1966); Lakoff and Johnson (1980); Lakoff and Núñez (2001).

^2^Cf. Merleau-Ponty (2012). In chap. IV of the Introduction, entitled “The Phenomenal Field,” he explains the vital communication with the world that we are given via sensation and perception.

^3^Gallagher and Zahavi show that Sartre also shares with Merleau-Ponty the idea of being one's own body, rather than possessing it; cf. Sartre (1956) and Merleau-Ponty (2012). In this work he affirms: “But I am not in front of my body, I am in my body, or rather I am my body.”

^4^For a more detailed analysis, see Kelly (2007).

^5^These ideas, which were already present in Husserl's thought (1970), are taken up by authors such as Noë (2004); Gibbs (2006).

^6^This concept deserves a treatment that I cannot give it in this article, especially after the appearance in 1999 of the book Naturalizing Phenomenology.
