# Erratum on: Habit and embodiment in Merleau-Ponty

**DOI:** 10.3389/fnhum.2015.00226

**Published:** 2015-04-22

**Authors:** Frontiers Production Office

**Affiliations:** Frontiers Production Office, FrontiersSwitzerland

**Keywords:** habit, Merleau-Ponty, embodiment, pre-reflective knowledge, Gallagher, Zahavi

Reason for Erratum:

Due to a typesetting error a section of the last sentence was misplaced, the final sentence is corrected to the following:

In contrast, they hold that there is a reciprocal influence between science and phenomenology, just as Varela et al. (1991) understood it via his neurophenomenology based on aspects of the phenomenology of perception of Merleau-Ponty (cf. Gallagher and Zahavi, 2008; see also Gallagher, 1997).

Footnote 6 is also corrected as follows:

^6^This concept deserves a treatment that I cannot give it in this article, especially after the appearance in 1999 of the book Naturalizing Phenomenology.

Minor grammatical errors were corrected. The aforementioned errors do not change the scientific conclusions of the article in any way. The original article was updated and the publisher apologizes for the mistakes.
